# *In Vitro* Activities of Kissorphin, a Novel Hexapeptide KiSS-1 Derivative, in Neuronal Cells

**DOI:** 10.1155/2012/691463

**Published:** 2012-07-09

**Authors:** Nathaniel G. N. Milton

**Affiliations:** Department of Human and Health Sciences, School of Life Sciences, University of Westminster, London W1W 6UW, UK

## Abstract

The primary products of the metastasis-suppressor KiSS-1 gene are the kisspeptin (KP) peptides that stimulate gonadotrophin-releasing-hormone (GnRH) release via GPR-54 receptor activation. Recent studies have suggested that the KP-10 peptide also activates neuropeptide FF (NPFF) receptors. The aim of the current study was to determine the activities of the KiSS-1 derivative kissorphin (KSO), which contains the first six amino acids of the KP-10 peptide, is C-terminally amidated, and shares amino acid similarities with the biologically active NPFF 3–8 sequence. The KSO peptide inhibited forskolin-stimulated cyclic adenosine monophosphate (cAMP) production in ND7/23 neuroblastoma cells via an action that could be inhibited by the NPFF receptor antagonist RF9. Release of GnRH by LA-N-1 neuroblastoma cells was not altered by the KSO peptide. In ND7/23 neuroblastoma cells, the KSO peptide was able to reduce forskolin neuroprotection against H_2_O_2_ toxicity. The KSO peptide was also able to prevent prostaglandin E2-induced apoptosis in rat cortical neurons. The NPFF receptor antagonist RF9 could inhibit these actions of the KSO peptide in oxidative stress and apoptosis models. In conclusion, the kissorphin peptide, comprising the amino acid sequence Tyr-Asn-Trp-Asn-Ser-Phe-NH_2_, has NPFF-like biological activity without showing any GnRH releasing activity and inhibits forskolin-activated cAMP release.

## 1. Introduction

Recent studies have shown that kisspeptin (KP) peptides can activate the two neuropeptide FF (NPFF) receptors, NPFFR1 (GPR-147) and NPFFR2 (GPR-74) [1–3]. These actions of KP peptides can be blocked by NPFF receptor antagonists including RF9 [4]. The NPFF peptide is a ligand for both these receptors [5–7], and the biologically active NPFF 3–8 form [8] shows amino acid sequence similarities to KP peptides (Table 1).

The KP peptides themselves play a central role in the hypothalamic-pituitary-gonadal (HPG)-axis by acting on gonadotrophin-releasing hormone (GnRH) neurons to activate GnRH release [9]. The KP peptides are encoded by the metastasis-suppressor KiSS-1 gene and are ligands for the GPR-54 receptor [1, 10–12]. A range of KP peptides are found *in vitro* and *in vivo* with the largest 54 amino acid form corresponding to residues 68–121 of the KiSS-1 encoded pre-pro-KP [10]. Shorter derivatives of KP comprising the C-terminal 14, 13, and 10 amino acids have also been found in tissues and corresponding to residues 108–121, 109–121, and 112–121 of the KiSS-1 encoded pre-pro-KP [10, 13]. Biological activity of KP peptides requires the KP 112-121 sequence with an amidated C-terminus [10, 13, 14]. 

The biological activities of NPFF include antiopioid [8, 15], analgesic [16], anorexigenic [17, 18], cardiovascular [19, 20], adipocyte [21], and immune cell [22] effects. The biological activities of NPFF involve an action that can be measured as inhibition of forskolin-stimulated cyclic adenosine monophosphate (cAMP) production and are mediated via activation of the NPFFR1 or NPFFR2 receptors [23–25].

Cleavage of KP peptides to remove the amidated C-terminal tripeptide Leu-Arg-Phe-NH_2_ of the KP sequence by matrix metalloproteinase (MMP) enzymes abolishes the activation of GPR-54 by KP peptides [26]. However, the resultant peptides have a C-terminal glycine residue that could be alpha-amidated [27] and C-terminal alpha amidation is a feature of many biologically active neuropeptides. As such the MMP cleaved KP peptides could have an alternative biological activity. In view of the similarities between KP and NPFF sequences it is possible that the hexapeptide fragment of KP-10 containing residues 1–6 with a C-terminally amidated phenylalanine residue could have NPFF-like activity. The aim of the study has been to determine if the KP derivative Tyr-Asn-Trp-Asn-Ser-Phe-NH_2_, termed kissorphin (KSO), has biological activity and to determine whether KSO peptides have NPFF-like activities in neuronal cell culture-based assays.

## 2. Materials and Methods

### 2.1. Materials

Synthetic NPFF, KP, and KSO peptides were obtained from Bachem. Mouse neuroblastoma x rat neuron hybrid ND7/23 [28] and human LA-N-1 neuroblastoma [29] cell lines were obtained from the Health Protection Agency, Cell Culture Collection. Rat cortical neurons and cell culture media were obtained from Invitrogen. The cAMP EIA kits and other test chemicals were obtained from Sigma-Aldrich. 

### 2.2. Cell Cultures

Mouse neuroblastoma x rat neuron hybrid ND7/23 and human LA-N-1 neuroblastoma cells were routinely grown in a 5% CO_2_ humidified incubator at 37°C in a 1 : 1 mixture of Dulbecco's modified Eagle's medium and HAM's F12 with Glutamax (Invitrogen) supplemented with 10% foetal calf serum (FCS), 1% nonessential amino acids, penicillin (100 units/ml), and streptomycin (100 mg/ml) [30–32]. Primary cultures of rat cortical neurons were prepared from cryopreserved rat cortex neurons [33] isolated from day-18 Fisher 344 rat embryos and cultured according to the supplier instructions in Neurobasal/B27 medium.

### 2.3. Inhibition of Forskolin Stimulated cAMP Assays

ND7/23 cells were plated onto 24-well plates in normal growth medium and cultured overnight. The medium was replaced with serum-free medium containing 100 *μ*M IBMX on the day of the experiment for 2 h prior to the addition of test compounds [3, 34]. Cells were then treated with medium alone (control) or medium containing 10 *μ*M forskolin and 100 *μ*M IBMX and varying doses (10 nM–100 *μ*M) of the synthetic NPFF, KP, or KSO peptides. Cells were then incubated at 37°C for a total of 30 min, after which the medium was aspirated and replaced with a 1 ml of 0.1 M HCl and incubated for 15 min to stop the reaction and lyse the cells. Cell lysates were centrifuged to remove cellular debris and cAMP levels in the supernatant determined. The ir-cAMP levels were determined using a direct enzyme immunoassay kit according to the manufacturers instructions. Samples and standards were acetylated prior to incubation with anti-cAMP antibody and cAMP-alkaline phosphatase conjugate. 

### 2.4. GnRH Release Assay

LA-N-1 cells were plated onto 24-well plates in normal growth medium and cultured overnight. On the day of experiment, cells were then treated with medium alone (control) or medium containing varying doses (10 nM–10 *μ*M) of the synthetic NPFF, KP, or KSO peptides. Cells were then incubated at 37°C for a total of 2 hours, after which the medium was aspirated for determination of GnRH levels. The immunoreactive (ir) GnRH was measured by EIA using synthetic biotinyl-GnRH-I, GnRH-I standards, primary anti-GnRH antibody coated immunoplates, and detection with streptavidin labeled HRP and TMB substrate.

### 2.5. Forskolin Neuroprotection against Hydrogen Peroxide (H_2_O_2_) Toxicity

ND7/23 cells were plated onto 96-well plates in normal growth medium and cultured overnight. On the day of the experiment cells were treated with combinations of media alone (control), H_2_O_2_ (250 *μ*M) to induce toxicity, forskolin (10 *μ*M) to protect against H_2_O_2_ toxicity [35], and 10 *μ*M NPFF, KP, or KSO peptides. Cells were incubated for 2 hours prior to determination of cell viability.

### 2.6. Prostaglandin-E_2_ (PGE_2_) Toxicity

Rat cortical neurons were plated onto 96-well plates in normal growth medium. On the day of the experiment, cells were treated with media alone (control), PGE_2_ (25 *μ*M) to induce toxicity [36], and either 10 *μ*M NPFF, KP, or KSO peptides. Cells were incubated for 24 hours prior to determination of cell viability.

### 2.7. Cell Viability

After treatment with test peptides or drugs and incubation for the appropriate time the viability was determined by either trypan blue dye exclusion with at least 100 cells counted per well or by MTT reduction [37]. For MTT reduction determination, after incubation with test substances MTT (10 *μ*l  : 12 mM stock) was added and cells incubated for a further 4 hours. Cell lysis buffer (100 *μ*l/well; 20% (v/v) SDS, 50% (v/v) N,N-dimethylformamide, and pH 4.7) was added and after repeated pipetting to lyse cells the MTT formazan product formation was determined by measurement of absorbance change at 570 nm. Control levels in the absence of test substances were taken as 100%, and the absorbance in the presence of cells lysed with Triton X-100 at the start of the incubation period with test substances was taken as 0% [38].

### 2.8. Data Analysis

All data are expressed as means ± s.e.m. For irGnRH measurements levels in samples were determined from a standard curve using synthetic GnRH-I as the standard. For cytotoxicity experiments data are expressed as % dead (trypan blue stained) cells or % control cells (MTT reduction). The significance of differences between data was evaluated by one-way analysis of variance (ANOVA). A *P* value of <0.05 was considered statistically significant.

## 3. Results

### 3.1. Effect of KSO Peptides on NPFF-Like Biological Activity

**Figure 1:**
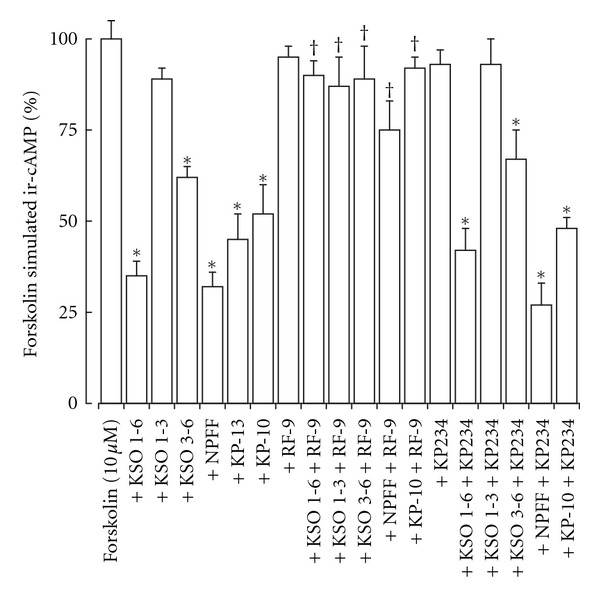
Effect of KSO, NPFF, and KP peptides on forskolin-stimulated ir-cAMP release from ND-7/23 cells. Cells were exposed to forskolin with KSO, NPFF, or KP peptides for 30 min prior to termination of the reaction with 0.1 M HCl. The effects of the NPFF receptor antagonist RF9 and the GPR-54 antagonist KP234 on KSO, NPFF, or KP peptide inhibition of forskolin-stimulated ir-cAMP release were also determined. Levels of ir-cAMP in the cell lysates were measured by EIA. Results are mean ± sem (*n* = 8). *P < 0.05 versus forskolin alone; ^†^ *P* < 0.05 versus KSO, NPFF, or KP peptide alone; one-way ANOVA.

The NPFF-like biological activity was determined by measurement of forskolin-stimulated ir-cAMP release in response to KSO, KP, and NPFF peptides. The KSO peptides containing residues 1–6 and 3–6 significantly inhibited forskolin stimulated ir-cAMP release from ND-7/23 cells (Figure 1), whilst the KSO 1-3 fragment had no effect. The NPFF, KP-10, and KP-13 peptides also significantly inhibited forskolin stimulated ir-cAMP release, which could be antagonized by the NPFF receptor antagonist RF-9 [4] but not the GPR-54 receptor antagonist KP234 [39]. The RF-9 and KP234 had no effect on forskolin-stimulated ir-cAMP release.

### 3.2. Effect of KSO Peptides on KP-Like Biological Activity

**Figure 2:**
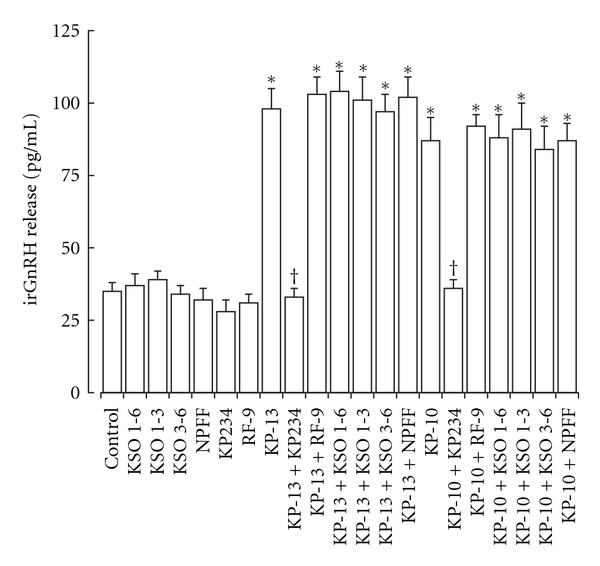
Effect of KSO, NPFF, and KP peptides on irGnH release from LA-N-1 neuroblastoma cells. Cells were exposed to KSO, NPFF, KP, the NPFF receptor antagonist RF9, and the GPR-54 antagonist KP234 for 2 hours. Levels of irGnRH in the media were measured by EIA. Results are mean ± s.e.m. (*n* = 8). *P < 0.05 versus control (media alone); ^†^ *P* < 0.05 versus KP peptide alone; one-way ANOVA.

The KP-like biological activity was determined by measurement of irGnRH release in response to KSO, KP, and NPFF peptides. The KSO peptides containing residues 1–6, 1–3 and 3–6 had no effect on irGnRH release from LA-N-1 neuroblastoma cells (Figure 2). The KP-10 and KP-13 peptides both stimulated a significant increase in irGnRH release, which could be antagonized by the GPR-54 receptor antagonist KP234 [39] but not the NPFF receptor antagonist RF-9 [4]. The KSO peptides had no effect on KP stimulated irGnRH release. NPFF, RF-9, and KP234 had no effect on irGnRH release.

### 3.3. Effect of KSO Peptides on H_2_O_2_ and PGE_2_ Neurotoxicity

**Figure 3:**
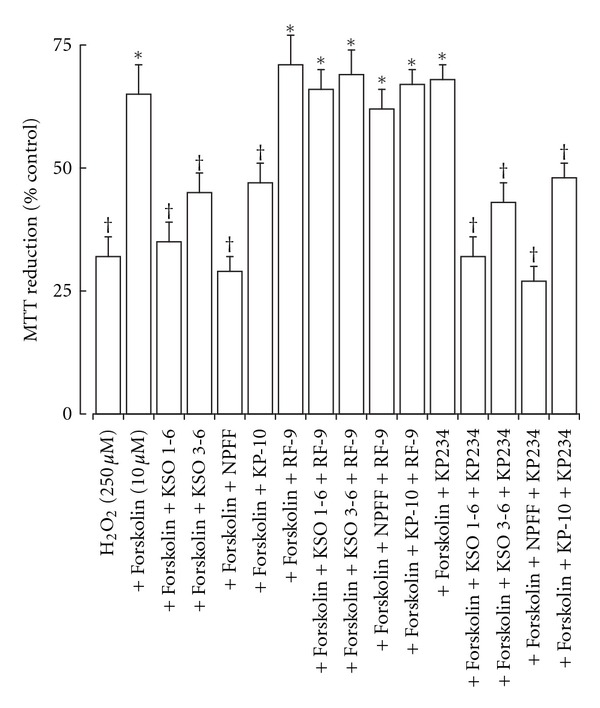
Effect of KSO, NPFF, and KP peptides on forskolin prevention of H_2_O_2_ toxicity in ND-7/23 cells. Cells were exposed to H_2_O_2_ either alone or in the presence of forskolin with KSO, NPFF, or KP peptides for 2 hours prior determination of cell viability by MTT reduction. The effects of the NPFF receptor antagonist RF9 and the GPR-54 antagonist KP234 were also determined. Results are expressed as % Control MTT reduction (mean ± sem; *n* = 8). *P < 0.05 versus H_2_O_2_ alone; ^†^ *P* < 0.05 versus H_2_O_2_ plus forskolin; one-way ANOVA.

**Figure 4:**
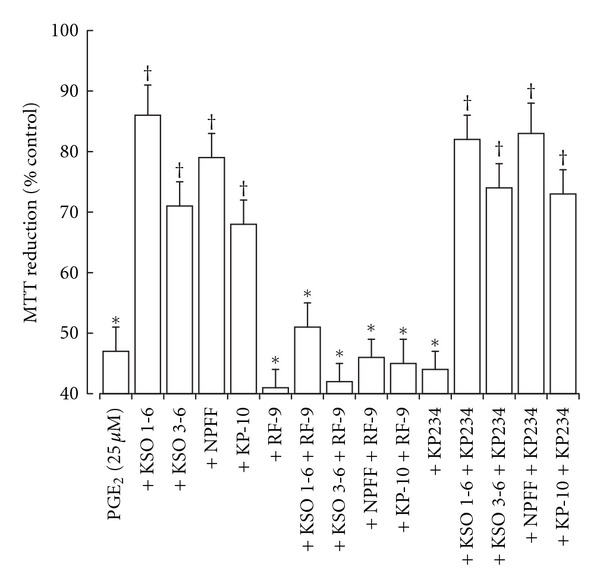
Effect of KSO, NPFF, and KP peptides on PGE_2_ toxicity in rat cortical neurons. Cells were exposed to PGE_2_ either alone or in the presence of KSO, NPFF, or KP peptides for 24 hours prior determination of cell viability by MTT reduction. The effects of the NPFF receptor antagonist RF9 and the GPR-54 antagonist KP234 were also determined. Results are expressed as % Control MTT reduction (mean ± s.e.m.; *n* = 8). *P < 0.05 versus control; ^†^ *P* < 0.05 versus PGE_2_ alone; one-way ANOVA.

The effects of KSO peptides were tested in two models of neurotoxicity. In the H_2_O_2_-stimulated-toxicity model the KSO 1–6 and 3–6 peptides, plus NPFF and KP-10 peptides, inhibited the forskolin protection against H_2_O_2_ toxicity in ND-7/23 cells (Figure 3). These effects could be reversed by the NPFF receptor antagonist RF-9 but not the GPR-54 antagonist KP234. In the PGE_2_-induced-toxicity model the KSO 1-6 and 3-6 peptides, plus NPFF and KP-10 peptides, were able to reduce the PGE_2_ toxicity (Figure 4). These effects could be reversed by the NPFF receptor antagonist RF-9 but not the GPR-54 antagonist KP234.

## 4. Discussion

The results show that the KSO peptide has biological activity in neuronal cell models that can be antagonized by the NPFF receptor antagonist RF-9 [4] but not the GPR-54 receptor antagonist KP234 [39]. The actions of KSO involve inhibition of stimulated cAMP release and are similar to documented effects for both NPFF [23, 40] and KP peptides [1–3] *in vitro*. The NPFF receptors respond to a number of RF-amide containing peptides [41], but this is the first demonstration of a response to an SF-amide peptide. The contribution of NPFFR1 and NPFFR2 receptors to the observed effects has not been tested. Both these receptors react with NPFF [42] and KP peptides [2, 3], and it is likely that KSO peptides will act on both receptor types. The cross-reactivity of NPFF with some KP antibodies [43] suggests shared tertiary structure similarity as well as sequence similarity exists. The ability of NPFF, KP, and KSO peptides to act in similar neuronal models suggests that the KSO sequence represents the region of KP with NPFF similarity. The distribution of NPFF receptors [23, 44–47] suggests that the main effects of KSO peptides is likely to be restricted and there may also be species differences. The specificity of the NPFF antagonist RF-9 [3, 4] used in this study does not rule out actions via other related receptors. However, the actions of KSO are shared with NPFF and KP peptides, suggesting that a common feature that RF-9 can block mediates the effects. Cell lines overexpressing NPFFR1 and NPFFR2 receptors have been used to determine receptor-binding affinities and cAMP inhibition by NPFF related peptides [6, 24, 42] and could be employed to confirm KSO interactions with NPFF receptors.

The ability of MMP enzymes to cleave KP-10 *in vitro* [26] to yield the KSO sequence, with an extra C-terminal Gly residue that can be amidated [27], raises the possibility that KSO-like peptides could be generated both *in vitro* and *in vivo*. The observations that KP peptides down regulate the expression of MMP enzymes [48] suggest that this action may be limited by release of KP peptides. However, there are conditions where MMP enzymes are elevated, and since the effects of KP peptides are at the level of protein expression rather than activity, there may be conditions where KSO-like peptides could be generated. The KSO sequence and MMP cleavage sites in the KiSS-1 precursor protein are shared between the human [10, 13] and rat forms [49]. It cannot be excluded that the actions of KP-10 and KP-13 in the cell models tested are due to generation of KSO-like peptides.

The ability of KSO peptides to prevent PGE_2_ toxicity in rat cortical neurons raises the possibility that KSO peptides could be neuroprotective; however, the reversal of forskolin neuroprotection by KSO peptides suggests that any neuroprotective properties may be model specific. The expression of appropriate NPFF or related receptors that KSO peptides can bind to and activate responses from may also be key for neuroprotective activity. In a recent study the KP peptides including the KSO 1–6 fragment were shown to be neuroprotective against amyloid peptides via a receptor-independent mechanism [50]. The results with the reversal of forskolin neuroprotection suggest that *in vivo* the KSO peptides could have multiple actions and therefore not show measurable neuroprotective properties. The requirement for doses in the *μ*M range suggests that the effects being observed may not be seen at physiological concentrations of endogenous KSO peptides.

The lack of GnRH releasing activity of the KSO peptides is in agreement with previous studies [26]. The KSO peptide used in the present study has an amidated C-terminus instead of the glycine residue used in the Takino et al. [26] study, however, this has not added a GnRH releasing bioactivity.

## 5. Conclusions

The present study has identified a biological activity as an inhibitor of stimulated cAMP release for the KSO peptides with the sequences Tyr-Asn-Trp-Asn-Ser-Phe-NH_2_ and Trp-Asn-Ser-Phe-NH_2_, which can be antagonized by an NPFF receptor antagonist. These actions are seen in neuronal cell models based on human, mouse, and rat cells, suggesting that the action is not species specific.

## Figures and Tables

**Table 1:** Comparison of kisspeptin, kissorphin, and NPFF sequences.

Kisspeptin-13	Leu	Pro	Asn	Tyr	Asn	Trp	Asn	Ser	Phe	Gly	Leu	Arg	Phe	NH_2_
Kisspeptin-10				Tyr	Asn	Trp	Asn	Ser	Phe	Gly	Leu	Arg	Phe	NH_2_
Kissorphin				Tyr	Asn	Trp	Asn	Ser	Phe	NH_2_				
Neuropeptide FF		Phe	Leu	Phe	Gln	Pro	Gln	Arg	Phe	NH_2_				
				^ ∗^	^ †^	^ ∗^	^ †^		^ ∗^					

^∗^Ring structure aminoacid; ^†^uncharged polar aminoacid.
